# Barriers and Enablers in Implementing Technology-Enabled Care for Older Adults in Rural and Remote Settings: A Scoping Review

**DOI:** 10.3390/ijerph23060713

**Published:** 2026-05-27

**Authors:** Michelle A. Krahe, Stephanie Baker, Emma Anderson, Edward Strivens, Sarah L. Larkins

**Affiliations:** 1 College of Medicine and Dentistry, James Cook University, Douglas, QLD 4811, Australia; emma.anderson1@jcu.edu.au (E.A.); edward.strivens@health.qld.gov.au (E.S.); sarah.larkins@jcu.edu.au (S.L.L.); 2 Northern Australian Regional Digital Health Collaborative, Douglas, QLD 4811, Australia; 3 College of Science and Engineering, James Cook University, Douglas, QLD 4811, Australia; stephanie.baker@jcu.edu.au; 4 Cairns and Hinterland Hospital and Health Service, Cairns North, QLD 4870, Australia

**Keywords:** technology-enabled care, telehealth, telemedicine, digital health, older adults, rural and remote health, implementation science

## Abstract

**Highlights:**

**Public health relevance—How does this work relate to a public health issue?**

- Limited access to healthcare, rehabilitation, and self-management support for older adults in rural, regional, and remote settings contributes to avoidable inequities in health outcomes.
- Technology-enabled care offers a mechanism to improve reach and continuity of care, yet implementation barriers constrain its public health impact in geographically dispersed communities.

**Public health significance—Why is this work of significance to public health?**

- It identifies theory-informed determinants of implementation using the Consolidated Framework for Implementation Research, highlighting capability, adaptability, and calibrated complexity as central drivers of uptake.
- It demonstrates that implementation depends less on technological sophistication alone than on alignment with local system capacity, workforce capability, and user needs.

**Public health implications—What are the key implications or messages for practitioners, policymakers and/or researchers in public health?**

- Interventions for older adults in rural and remote areas should minimize cognitive and technical burden, embed structured training, and leverage relational support to strengthen adoption and sustainment.
- Policy and investment decisions should prioritize infrastructure, workforce capability, and context-sensitive design to ensure technology-enabled care delivers equitable population-level benefit.

**Abstract:**

Existing reviews of technology-enabled care for older adults have primarily focused on technology usability, patient acceptance, and clinical outcomes. However, there remains limited synthesis of the organizational and system-level factors influencing the implementation of technology-enabled care in rural, regional, and remote contexts. This review addresses this gap by mapping barriers and facilitators using the Consolidated Framework for Implementation Research. Using technology to enable or enhance healthcare, rehabilitation, and self-management offers significant potential to improve access, outcomes, and equity for older adults; however, adoption and sustained use in rural, regional, and remote (RRR) settings remain limited. This scoping review aimed to identify factors influencing the implementation of technology-enabled care interventions for community-dwelling older adults in RRR contexts. Searches were conducted in PubMed, MEDLINE, CINAHL, Web of Science, and Scopus for empirical studies published from 2014 onwards. Barriers and enablers were mapped to the Consolidated Framework for Implementation Research (CFIR) and synthesized narratively. The search identified 807 records, of which 433 remained after duplicate removal and 105 proceeded to full-text assessment. Five studies met the inclusion criteria, examining telehealth, telerehabilitation, remote monitoring, and mobile health applications. Across the included studies, 71 implementation factors were identified, comprising 39 barriers and 32 enablers mapped across five CFIR domains and 20 constructs. The most frequently reported barriers related to innovation recipients’ capability, innovation design, innovation complexity, and outer setting local conditions. The most frequently reported enablers related to innovation adaptability, innovation complexity, and innovation recipients’ motivation. Findings suggest that implementation in RRR settings depends less on technological sophistication than on aligning design and delivery with user capability and local system capacity, reducing cognitive and technical burden, and embedding relational and contextual support.

## 1. Introduction

Technology-enabled care is an increasingly prominent approach to challenges facing contemporary healthcare. It involves the convergence of health systems enhanced by digital and other enabling technologies, which allow access to data and information more easily [1]. These include telehealth and telerehabilitation, remote monitoring, digitally supported self-management, assistive technologies, and hybrid models integrating technology with in-person or community-based care [2,3]. Collectively, such approaches aim to extend service reach, improve continuity and coordination, and empower individuals to actively manage their health, functional capacity, and wellbeing. For health systems facing growing demand, workforce shortages, and fiscal pressures, technology-enabled care offers the potential to improve access and equity while enabling more efficient and flexible service delivery.

Across 27 OECD countries, an average of 58% of adults aged 65 or over reported living with two or more chronic diseases, with rates exceeding 70% in some nations [4]. In Australia, 80% of Australians aged 65 and over have at least one chronic health condition, and 28% have three or more, indicating high multimorbidity in later life [5]. Among this age group, dementia, coronary heart disease and chronic obstructive pulmonary disease are leading contributors to overall burden. Evidence suggests that, when appropriately designed and implemented, technology-enabled care can improve health outcomes, functional ability, and self-management capacity, particularly for people with chronic or complex conditions [6,7]. For community-dwelling older adults, technology-enabled approaches have been associated with reduced travel burden, enhanced access to specialist care, and greater opportunities to age well in place [8,9,10]. However, the successful adoption of such interventions depends not only on technological capability but also on the ways the interventions are introduced, integrated, and sustained within routine care. Implementation processes, including alignment with local service models, workforce roles, organizational routines, and broader system conditions, can play a critical role in determining whether technology-enabled care translates into meaningful and equitable outcomes [11,12].

Digital health implementation research increasingly recognizes that adoption and sustainability are shaped by complex interactions between technologies, users, organizations, and wider systems. Several theoretical perspectives have been applied to examine these dynamics. For example, the NASSS Framework highlights the complexity of implementing technology-enabled care across seven interacting domains, including the technology, value proposition, adopters, organizational context, and wider system conditions [13,14]. Similarly, models derived from Technology Acceptance Model research emphasize how perceived usefulness, ease of use, and user attitudes influence the uptake of digital tools among both clinicians and patients [15]. In parallel, socio-technical systems theory underscores the interdependence between technological artifacts and the social, organizational, and workflow environments in which they are embedded [16]. Collectively, this body of work demonstrates that successful implementation of technology-enabled care requires attention not only to the technical performance of digital interventions but also to the organizational routines, workforce capabilities, governance structures, and contextual conditions that shape everyday use.

These considerations are particularly salient for people living in rural, regional, and remote (RRR) settings, where longstanding inequities in healthcare access persist [17,18,19]. Geographic distance, limited service availability, fragmented care pathways, and persistent workforce shortages frequently constrain the timeliness, continuity, and responsiveness of care [20,21]. In these contexts, technology-enabled care has been widely promoted as a mechanism to extend service reach, support care closer to home, and enable older adults to maintain independence and quality of life. Policy investments during and following the COVID-19 pandemic have further accelerated uptake, often at scale and under conditions of rapid implementation.

Much of the existing literature focuses on feasibility, usability, or clinical outcomes, with limited attention given to the organizational, workforce, and relational conditions needed to adapt and embed these services into routine community-based care [19,22,23]. Commonly reported challenges in other populations and settings include limited digital capability, usability constraints, safety concerns, workflow disruption, resistance to change, funding limitations, and inadequate infrastructure. However, it remains unclear which determinants are most influential for older adults in RRR contexts or how these factors interact with local social, cultural, and environmental characteristics. Several reviews have examined the adoption of technology-enabled care among older adults, often focusing on usability, acceptability, or clinical outcomes [24,25,26,27,28,29,30]. For example, reviews have explored barriers and facilitators to technology adoption among older adults with chronic diseases, identifying factors such as digital literacy, perceived usefulness, and social support as key influences on uptake [28]. Other reviews have examined the use of assistive technologies and telehealth among older populations, highlighting barriers such as usability challenges, privacy concerns, and limited awareness of available technologies [29,30]. While these syntheses provide valuable insights into user experiences and technology acceptance, they typically focus on individual-level determinants or specific technologies. Less attention has been given to the organizational, workforce, and system-level conditions that shape the implementation and sustainability of technology-enabled care, particularly within RRR contexts.

RRR settings introduce distinctive challenges and opportunities for digital health implementation, including workforce shortages, constrained infrastructure, variable digital connectivity, and reliance on cross-sector and community-based service models [31,32]. These contextual characteristics may influence not only whether technology-enabled care can be adopted, but also how it is adapted, coordinated, and sustained over time [33]. Despite increasing emphasis on digital health to address geographic inequities, there remains limited synthesis of implementation determinants specifically affecting technology-enabled care for community-dwelling older adults in RRR environments [12,13,34].

Many reviews of technology-enabled care for older adults have focused on usability, acceptability, adoption, clinical outcomes, or specific technologies, and therefore tend to foreground individual-level factors such as digital literacy, perceived usefulness, confidence, and user experience. While these issues are important, they provide limited insight into the organizational, workforce, relational, and system conditions that determine whether technology-enabled care can be embedded and sustained in RRR settings. This distinction is important because barriers such as workforce capacity, service integration, infrastructure, local adaptation, and support roles are not simply background context; they are implementation determinants that shape whether digital health interventions can operate equitably and sustainably outside metropolitan environments.

**Figure 1:**
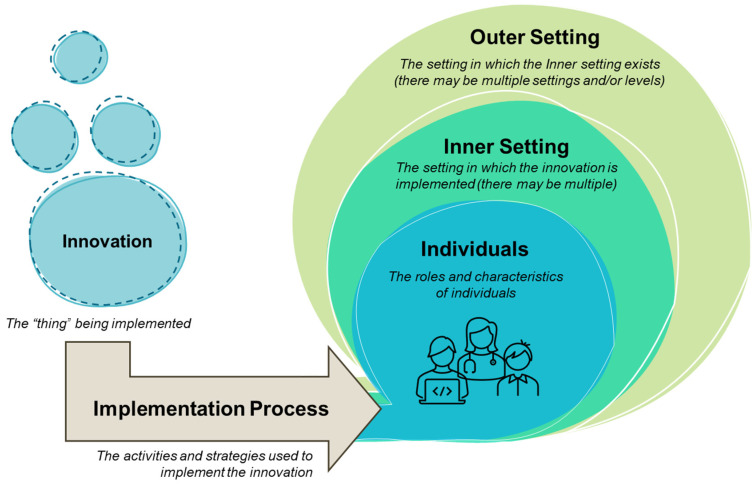
The five domains of the CFIR. Adapted from [12].

To address this gap and support a theoretically informed synthesis, this review used the Consolidated Framework for Implementation Research (CFIR) [35,36,37], a widely used determinant framework in implementation science. The framework comprises 39 constructs organized across five domains: innovation, outer setting, inner setting, individuals, and implementation process (Figure 1). While frameworks such as the NASSS Framework and Technology Acceptance Model provide important insights into technology adoption and complexity, the CFIR offers a comprehensive structure for examining determinants operating across intervention characteristics, organizational settings, individual actors, and implementation processes. This breadth makes it well suited for synthesizing heterogeneous evidence across diverse technology-enabled care interventions [38].

In this scoping review, we aim to identify barriers and facilitators to implementing technology-enabled care interventions for community-dwelling older adults in RRR areas. By systematically mapping existing evidence, we aim to clarify what is known, identify gaps, and inform policymakers, service leaders, and researchers in designing, delivering, and scaling technology-enabled care models responsive to the realities of aging in non-metropolitan settings.

## 2. Materials and Methods

### 2.1. Search Strategy

We conducted this review in accordance with the Preferred Reporting Items for Systematic Reviews and Meta-Analyses Extension for Scoping Reviews (PRISMA-ScR) guidelines (Table S1) [39,40]. We selected a scoping review approach to comprehensively map existing evidence on technology-enabled care interventions for community-dwelling older adults living in RRR settings. This methodology is particularly well suited to areas where evidence is heterogeneous, conceptually diffuse, or emerging, and where understanding implementation conditions is as critical as understanding service outcomes.

We searched PubMed, MEDLINE (Ovid), CINAHL (EBSCO), Web of Science, and Scopus for peer-reviewed articles. The full search strategy for each database is provided in Supplementary File S1. An initial search in MEDLINE (Ovid) helped calibrate the syntax, identify relevant search terms, and subject headings, and test combinations across the key concepts of “technology-enabled care”, “older adults”, and “RRR settings”. We also screened reference lists of all included studies to identify additional relevant sources. Searches were limited to articles published between January 2014 and September 2025. We included primary empirical studies (including quantitative, qualitative, or mixed methods) and reviews, protocols, conference abstracts, commentaries, and other non-empirical sources were excluded. The review search strategy was registered with the Open Science Framework (https://doi.org/10.17605/osf.io/mn5hj).

### 2.2. Study Selection

Two reviewers (MAK and SB) independently screened all titles and abstracts in a two-stage process comprising (1) title and abstract screening and (2) full-text assessment. A third reviewer (EA) independently assessed studies flagged as unclear, along with an additional 10% subsample, to ensure consistency and resolve disagreements by consensus. Reasons for exclusion at the full-text stage were documented and detailed in a PRISMA flow diagram [41]. Eligibility for this review was defined using the Population, Concept, and Context framework.

We included studies that focused on older adults aged 65 years or older or studies reporting a mean or median age of 65 years or above (population). The age threshold for Indigenous participants was lowered to 50; however, none of the included studies provided disaggregated data for this group. We considered only community-dwelling adults; hospital-based or residential aged-care studies were excluded unless explicitly linked to technology-enabled services delivered in the community.

We included studies examining technology-enabled care (concept), defined as healthcare, rehabilitation, or self-enablement delivered remotely or integrated with other care. Eligible technologies included telehealth, telerehabilitation, remote monitoring, mobile health applications, digital self-management tools, and virtual or augmented modalities. We excluded studies focused on technology acceptance, device testing, or algorithm development without applied care delivery.

We focused on studies in RRR settings within high-income countries (context). Because rurality is defined differently across jurisdictions, we applied a context-sensitive classification approach. Where reported, rurality was classified using national or jurisdictional rurality frameworks (i.e., Modified Monash Model in Australia [42]). In other cases, we relied on the authors’ descriptions of the service context. Studies conducted exclusively in metropolitan settings or mixed metropolitan and RRR settings were excluded. Studies conducted in mixed geographic settings were considered only when findings specific to rural or remote populations could be clearly identified. Studies where the geographic context could not be clearly determined were excluded. As a result, a number of studies identified through the search were excluded at full-text screening because they did not explicitly report or demonstrate a RRR setting. Studies published in English, peer-reviewed, and full-text accessible were included.

### 2.3. Data Extraction

We imported search results into EndNote 20 (Clarivate, London, UK) and removed duplicates using automated and manual processes. Data were charted using a structured extraction form developed in Microsoft Excel (Microsoft, Redmond, WA, USA) and iteratively refined the form to ensure clarity and comprehensive coverage of information relevant to the review objectives. One reviewer charted all data, and a second reviewer verified entries to ensure accuracy and completeness. Data extraction was conducted using a structured form capturing study characteristics, intervention type, setting, and reported barriers and enablers to implementation. Identified determinants were subsequently mapped to constructs within the CFIR. Coding was conducted iteratively, with constructs reviewed and refined to ensure consistent interpretation across studies. Where uncertainties or disagreements arose during coding, these were resolved through discussion among the research team to reach consensus. Because this review aimed to map the evidence base rather than assess intervention effectiveness, we did not undertake a critical appraisal of study quality.

### 2.4. Data Synthesis

We used a framework synthesis approach, with Damschroder et al.’s CFIR as the primary coding framework [43]. Each barrier and enabler was coded according to the relevant CFIR domain and construct using the publicly available CFIR codebook template. Findings are presented as a descriptive narrative synthesis supported by visualization of mapped barriers and enablers.

## 3. Results

**Figure 2:**
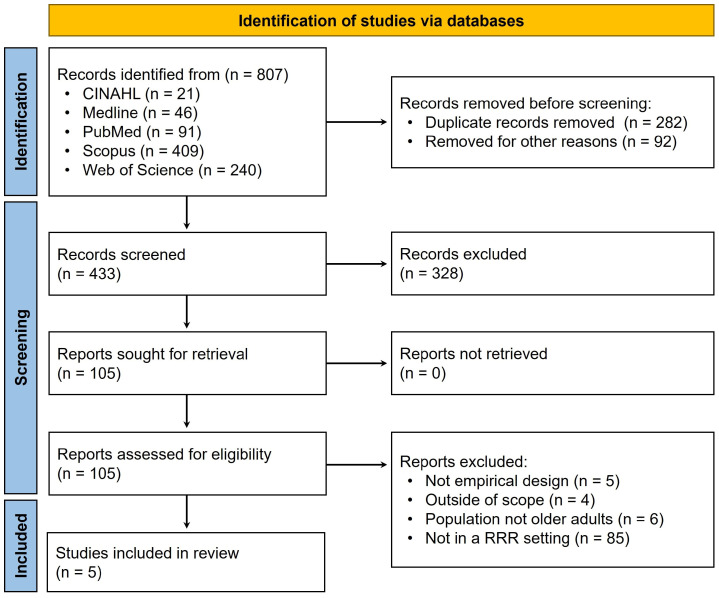
The PRISMA flow diagram illustrates the sequential process by which studies were identified, screened, assessed, and included in this review [41].

The literature search identified 807 articles. After removing duplicates, we screened 433 articles based on title and abstract, which yielded 105 articles for full-text screening. Because many abstracts did not clearly report whether studies were conducted in RRR settings, records were carried forward where eligibility could not be confidently determined. This cautious screening approach reduced the risk of prematurely excluding potentially relevant studies but resulted in a high number of setting-related exclusions at full-text review. We excluded articles based on ineligible study design or scope (*n* = 9, 8.6%), population (*n* = 6, 5.7%), and setting (*n* = 85, 81.0%). Five studies were included in the qualitative synthesis, and reference checking did not identify any additional studies. Figure 2 presents the study selection process.

### 3.1. Study Characteristics

Table 1 summarizes the characteristics of the included studies, published between 2016 and 2025; three originated from the United States [44,45,46] and one each originated from China [47] and Australia [48]. The three studies from the United States were situated in rural and underserved community settings and emphasized telemedicine access, community health worker involvement, and patient-centered chronic disease management. The study from China was conducted in rural, resource-poor settings and emphasized local contextualization of mobile health messages to language, literacy, and delivery preferences. The Australian study was conducted in a regional context and involved home-based telemonitoring supported by telehealth nursing and broadband-enabled infrastructure.

Collectively, the included studies aimed to examine the development, feasibility, usability, and preliminary effectiveness of digital and technology-enabled care interventions to support self-management among older adults with chronic conditions. The studies ranged from intervention development and contextualization, through feasibility and usability testing, to evaluations of patient-centered models integrating telehealth, community-based support, and remote monitoring to improve health behaviors, outcomes, and service use. The target population was community-dwelling older adults living with chronic conditions in rural or regional settings (*n* = 293). Health foci included secondary prevention and self-management following stroke, chronic disease prevention and lifestyle management, diabetes care among underserved older adults, multimorbidity management through home-based telemonitoring, or medication self-management in the context of polypharmacy. Collectively, the interventions focused on community- and home-based service models that leveraged telehealth or mobile technologies to support self-management, continuity of care, and patient–provider engagement, often integrating digital tools with primary care, allied health, nursing, or community health worker support.

### 3.2. Barriers and Enablers in Implementation

**Figure 3:**
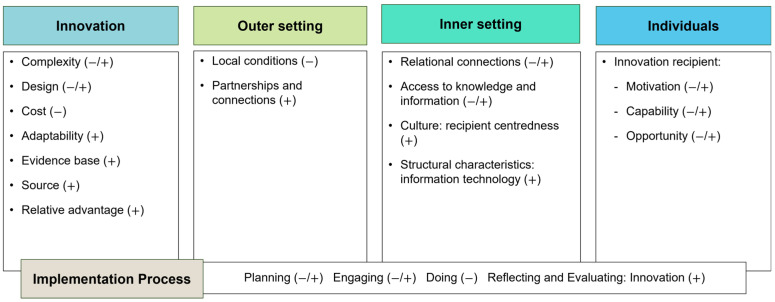
Barriers (−) and enablers (+) implementing technology-enabled care for older adults in RRR settings, as grouped by CFIR domains and constructs [36,37].

Across the included studies, 71 factors influencing the implementation of technology-enabled care interventions for community-dwelling older adults in RRR settings were identified. These factors were coded as barriers (*n* = 39) or enablers (*n* = 32) and mapped to the five CFIR domains and 20 CFIR constructs (Supplementary File S2; Figure 3). Three of the constructs mapped exclusively to barriers, ten to enablers, and nine reflected mixed valence. The most frequently reported barriers related to innovation recipients’ capability (10/39, 25.6%), innovation design (8/39, 20.5%), innovation complexity (5/39, 12.8%), and outer setting local conditions (4/39, 10.3%). These barriers reflected difficulties associated with digital and health literacy, cognitive or memory-related challenges, intervention usability, technical burden, and contextual constraints such as connectivity, infrastructure, and home support. The most frequently reported enablers related to innovation adaptability (5/32, 15.6%), innovation complexity (4/32, 12.5%), innovation recipients’ motivation (3/32, 9.4%), and constructs linked to design, evidence base, relative advantage, access to knowledge and information, and relational connections. Although the barriers and enablers are described using standard CFIR terminology, all findings are drawn from studies conducted in RRR settings, and the section below describes how these factors are experienced within this context.

#### 3.2.1. Innovation

The innovation domain featured in all five studies, particularly in relation to design (10/71; 14.0%), perceived complexity (9/71; 12.7%), and adaptability (5/71; 7.0%) of interventions. The studies most commonly linked barriers to design features that increased cognitive or technical burden, such as text-heavy or professionally framed content, insufficient tailoring to individual needs, complex interfaces, rigid scheduling requirements, and underutilized or poorly integrated features. Inconsistent message sources and high perceived costs further undermined trust and engagement. Conversely, innovation-related enablers stemmed from design choices that reduced burden and supported usability. These included voice-based delivery, use of plain language and local dialects, structured messaging focused on a single key point, repetition with adjustable playback speed, and optimization of timing and frequency to align with everyday routines. Across studies, simplicity of core features, short setup and download times, and accessible, user-friendly technologies consistently improved acceptability and uptake.

#### 3.2.2. Outer Setting

The outer setting appeared less frequently referenced as an influencing factor, but it captures external conditions beyond the control of the implementation team or delivery organization that shape participants’ engagement with technology-enabled care. Key barriers in this domain related to social conditions and home support systems. Participants living alone or with limited informal support had fewer opportunities for reinforcement, troubleshooting, or encouragement in using the technology. Variable involvement of family members or informal carers further reduced continuity of information sharing and adherence to program activities. The studies also highlighted broader structural constraints, including unstable internet connectivity and limited access to appropriate devices, which impeded participation. Technical eligibility requirements, such as the need for broadband or NBN access, further restricted who could engage with the intervention. Conversely, alignment with existing care pathways, such as telemonitoring data transmitted directly to participants’ general practitioners, enabled engagement by strengthening connections to the broader health system and embedding the intervention within routine clinical workflows.

#### 3.2.3. Inner Setting

Inner setting factors shaped implementation through organizational, relational, and workforce conditions. Barriers included the workforce burden associated with extensive training and supervision requirements, coordination challenges across multiple providers, and the labor-intensive nature of some delivery models. At the same time, strong relational connections, such as therapeutic relationships with participants (e.g., intervention delivery by trained occupational therapy practitioners), and interdisciplinary collaboration among health workers, nurses, physicians, and pharmacists, emerged as important enablers. An organizational culture prioritizing person-centered care, such as tailored sessions and collaborative goal setting, promoted engagement, motivation, and ownership of lifestyle changes [44]. Interventions supported by structured training, supervision, IT support, and adequate IT infrastructure achieved more consistent delivery [45].

#### 3.2.4. Individuals

Individual-level factors influenced implementation through capability, opportunity, and motivation of innovation recipients (19/71; 26.7%). Participants with limited digital or health literacy, cognitive decline or poor memory, or minimal prior experience with mobile applications or videoconferencing often struggled to read text-based content, navigate devices, or retain information. These challenges reduced confidence, comprehension, and independent use of technology-enabled care and were often compounded by time commitments, competing priorities, or discomfort with technology. Enablers included recipients with higher baseline health literacy, willingness to engage, self-selection into programs, and motivation supported through approaches such as motivational interviewing, appreciative inquiry, and positive psychology. Personal contact with healthcare providers, particularly via videoconferencing, further supported engagement and implementation.

#### 3.2.5. Implementation Process

Factors related to planning, delivery, and refinement within the implementation process shaped outcomes. Barriers included short recruitment time limits and late enrolment, labor-intensive pilots, and resource-heavy delivery models that constrained scalability and resulted in incomplete participation. Scheduling home visits and telehealth sessions required flexibility, while delivery by program developers raised questions about transferability to routine service contexts. Enablers included structured program planning supported by fidelity checklists, guided support through in-person instruction and practice with technology, and integration of pilot feedback to iteratively refine content and delivery.

## 4. Discussion

This review examines barriers and enablers influencing the implementation of technology-enabled care interventions for community-dwelling older adults in RRR settings, mapped to the CFIR. Although the small number of eligible studies highlights a limited evidence base, the findings provide preliminary insights into how implementation factors are reported across CFIR domains in these contexts. Factors relating to innovation design, complexity, recipient capability, and relational support were frequently reported as influencing implementation experiences.

The findings suggest that implementation challenges in RRR contexts may relate less to technological sophistication and more to how interventions are designed and delivered within local systems. In particular, studies described how design choices that reduce cognitive and technical burden, incorporate adaptability, and leverage relational support were associated with greater reported acceptability and engagement. Importantly, several factors operated as either barriers or enablers depending on how they were configured and supported within specific implementation contexts. This highlights the importance of considering interactions between intervention design, recipient capability, and service context when implementing technology-enabled care in RRR settings.

Recent transdisciplinary scholarship on demographic transformation argues that challenges associated with population aging, such as healthcare access, technology adoption, and care delivery, cannot be effectively addressed within disciplinary or sectoral silos but instead require coordinated approaches that integrate clinical care, technological innovation, social support systems, and policy environments [49]. This perspective aligns closely with the findings of the present review. Barriers and enablers identified across the included studies emphasized the importance of place-based co-design, relational support structures, contextual adaptation of technologies, and alignment with local service capacity. In RRR settings, where healthcare systems often operate with limited workforce and fragmented service ecosystems, the implementation of technology-enabled care may therefore represent not only an implementation science challenge but also a broader systems coordination challenge involving stakeholders across healthcare, community services, digital infrastructure, and governance.

### 4.1. Design as an Implementation Consideration

Findings from the included studies suggest that innovation design plays an important role in shaping implementation experiences in RRR contexts. Several studies reported barriers linked to design features that implicitly assumed higher levels of literacy, digital confidence, or familiarity with technology. Text-heavy communication, professionally framed language, and complex interfaces were associated with reduced comprehension, trust, and engagement among some participants. Studies also described design features intended to reduce cognitive and technical burden, including voice-based delivery, plain language, use of local dialects, structured messaging focused on single key points, and adjustable playback speeds. These features were reported to support comprehension and usability for some participants. Taken together, these findings suggest that design choices may influence not only usability but also who is able to meaningfully engage with technology-enabled care interventions in RRR settings [50,51].

### 4.2. Adaptability and Place-Based Implementation

Adaptability was frequently described as an enabling factor across studies, particularly where interventions allowed adjustments to timing, frequency, or mode of delivery. Flexible scheduling, multimodal delivery, and combinations of in-person and remote care were reported to help align interventions with participants’ routines and local service conditions. These observations contribute to ongoing discussions regarding fidelity and adaptation in implementation research [52]. Instead, adaptability preserves core intervention functions across diverse and resource-constrained contexts [53,54,55]. In the included studies, adaptations such as flexible scheduling, multimodal delivery, tailored support, and blended in-person/remote care appeared to help maintain core intervention functions while improving fit with local service conditions. However, few studies formally measured adaptation processes or fidelity outcomes. Therefore, these findings suggest that adaptability may support implementation fit in RRR settings, but further prospective evaluation is needed to determine how adaptation affects fidelity, effectiveness, and sustainability.

### 4.3. Recipient Capability and Relational Support

Recipient capability was reported across all included studies as an influence on implementation. Barriers included limited digital literacy, health literacy, cognitive decline, and minimal prior experience with technologies. These factors were often described as reducing confidence and independent engagement with technology-enabled care. Several studies highlighted the role of relational support in addressing these challenges. For example, community health workers or care providers sometimes provided in-home guidance, technical assistance, and encouragement, helping participants develop skills and confidence in using the technology [45]. These findings suggest that trusted interpersonal relationships may play an important facilitating role when implementing technology-enabled care among older adults in RRR communities. However, relying heavily on clinical or community care staff to provide digital support may have implications for the workforce [56,57]. Technical tasks such as device setup or troubleshooting may extend beyond existing scopes of practice and add to workloads in already resource-constrained settings. Alternative models, such as dedicated digital navigators or community-based digital inclusion initiatives, may complement healthcare roles and support implementation sustainability [58,59].

### 4.4. Complexity and Implementation Fit

Innovation complexity was reported across the included studies as influencing implementation experience. Interventions perceived as overly complex or disruptive to daily routines were associated with confusion, disengagement, or limited use of intervention features [60,61,62,63,64]. Even modest increases in perceived complexity may be amplified in RRR contexts characterized by variable connectivity, limited support, and dispersed populations [12]. Interventions designed around simple core functions were described as easier to adopt and maintain. These findings suggest that perceived complexity may interact with contextual conditions such as connectivity limitations, availability of support, and recipient capability. Designing interventions that prioritize essential functions while introducing additional features gradually may therefore support implementation in RRR contexts.

Although limited internet coverage is often considered a primary barrier in RRR technology-enabled care, it was not the most frequently reported challenge across the included studies. Several factors may explain this. Many interventions incorporated strategies to mitigate connectivity issues, including voice-based messaging compatible with low-bandwidth networks, provision of mobile Internet devices, and flexible accommodation of 3G/4G connections where fixed-line broadband was unavailable. Additionally, the included studies emphasized intervention design, recipient capability, and relational support, which were frequently reported as more proximal determinants of implementation success. These observations highlight that while Internet coverage is a critical consideration in RRR settings, its impact may be moderated by adaptive implementation strategies, flexible delivery models, and support mechanisms, reinforcing the importance of a systems-informed approach to intervention planning.

### 4.5. Co-Design and Capacity Building

Co-design or user engagement processes aimed at tailoring interventions to local service environments and participant needs were described. Involving intervention designers, care providers, and intended recipients was reported to support alignment with local workflows, infrastructure constraints, and social contexts [65,66]. However, co-design alone may not fully address capability barriers where digital literacy or health literacy is limited. Implementation strategies may therefore benefit from incorporating structured training, ongoing technical support, and skill-building opportunities for participants [59,67,68,69]. These approaches may help strengthen digital confidence and support sustained engagement with technology-enabled care interventions.

### 4.6. Barrier Mitigation Strategies

The included studies also provide insights into strategies used to mitigate barriers to implementation. Across contexts, interventions often incorporated multiple complementary approaches to support participant engagement and maintain delivery of care intervention functions. Strategies included adapting content delivery to local languages and literacy levels, simplifying messages to focus on one key point per communication, and standardizing delivery through consistent timing and sender identification. Voice messaging, slowed playback speeds, and verification by local healthcare providers were reported to enhance comprehension and trust. Flexibility in scheduling and session format allowed participants to engage according to their availability and may have reduced attrition. Person-centered coaching, goal setting, and screening for technology skills helped ensure participants could meaningfully interact with the interventions despite varying levels of digital or health literacy. Home visits, telemedicine support, and real-time supervision by trained community health workers or healthcare providers facilitated technical troubleshooting and reinforced engagement, particularly in locations with limited digital infrastructure. Implementation fidelity was supported through checklists, structured session delivery, and ongoing supervision. Together, these strategies illustrate that successful implementation in RRR settings relies not only on intervention design but also on proactive, contextually tailored support mechanisms that address participant, technological, and systems-level barriers.

### 4.7. Study Limitations

Several limitations affect the interpretation of these findings. The small number of included studies reflects the limited published evidence examining technology-enabled care implementation for older adults in RRR settings. As a result, the findings should be interpreted as indicative of emerging patterns rather than a comprehensive mapping of the evidence base. In addition, the geographic distribution of the included studies was limited, with three conducted in the United States, one in China, and one in Australia. Rural health systems vary substantially across countries in terms of infrastructure, workforce models, digital maturity, and policy contexts. As such, the findings may not fully capture implementation determinants relevant to other international settings and should be interpreted with this contextual variability in mind. The review also did not undertake formal double-independent data extraction, although extracted data and CFIR mapping were verified by a second reviewer.

We restricted the review to published, peer-reviewed English-language literature, which may introduce publication and language bias. Importantly, while the review focused on older adults, few included studies explicitly examined the implications of cognitive impairment or dementia for implementation. Given the high prevalence of cognitive decline in later life, this represents a significant gap. Cognitive impairment may influence digital literacy, capacity for informed consent, sustained engagement with technology, and reliance on informal carers or support workers. As such, implementation determinants identified in this review may not fully account for the additional design, training, relational, and governance considerations required to support older adults with cognitive decline. Despite these limitations, the review provides a structured theory-informed synthesis of implementation determinants relevant to technology-enabled care in RRR settings.

### 4.8. Future Directions

The findings also reinforce calls for transdisciplinary approaches to aging systems, where digital health innovation is developed and implemented through coordinated partnerships spanning healthcare providers, community services, digital infrastructure, and policy governance. From a policy perspective, these findings suggest that investment in digital health in RRR settings may need to consider implementation readiness alongside technological capability [58]. Factors such as workforce capacity, training, infrastructure support, and integration with existing care pathways will influence whether technology-enabled care can be effectively embedded in routine services. For practice, included studies highlight the importance of intervention design, adaptability, and relational support in shaping implementation experiences. Allowing time and resources for place-based adaptation and user engagement may support contextual fit within local service environments. While these studies did not provide direct evidence regarding sustainability beyond funded trial conditions, these factors suggest potential challenges for scaling interventions in resource-constrained RRR settings. Interpreted through an implementation science lens, sustainability in these contexts may require streamlined intervention designs, integration with existing workflows, ongoing technical support, and capacity-building strategies to reduce workforce burden. Future research should explicitly evaluate long-term feasibility, resource requirements, and scalability to ensure that technology-enabled care interventions remain effective and sustainable outside of controlled trial environments.

## 5. Conclusions

Implementation of technology-enabled care interventions for community-dwelling older adults in RRR settings remains an emerging area of research. The limited number of included studies highlights a substantial gap in the evidence base, particularly for implementation-focused research that examines organizational, workforce, and system-level determinants. Across the included studies, implementation appeared to be shaped by the fit between intervention complexity, older adults’ digital and health literacy, available relational support, and local service conditions. Interventions appeared more acceptable and usable when they reduced cognitive and technical burden, incorporated flexible and low-burden modes of delivery, and embedded support from trusted providers, community health workers, or other care relationships. These findings suggest that technology-enabled care in RRR settings should not be designed as a standalone digital solution but as a supported model of care that is calibrated to user capability, local workforce capacity, infrastructure constraints, and existing care pathways. Given the small evidence base, findings should be interpreted as indicative rather than definitive, and further research should examine sustainability and scale-up in routine RRR service contexts.

## Figures and Tables

**Table 1:** Characteristics of the included studies.

Author, Year, Country	Aim	Study Design	Population (Sample Size), Mean Age ± SD	Concept	Context	Primary Outcomes/Measures
Leverentz et al., 2025, United States [44]	To assess the feasibility and potential effectiveness of a brief, individually delivered telehealth lifestyle intervention for improving occupational performance and health-related quality of life in older adults.	Nonrandomized pilot study using a single-group, pre–post-test design	Community-dwelling older adults (*n* = 12) Range: 65 to 85 years	Individually delivered telehealth lifestyle intervention comprising weekly synchronous video sessions over six weeks. The intervention supported personalized goal setting and behavior change across multiple lifestyle domains using occupational therapy-led strategies.	Rural	Functional occupational performance and health-related quality of life
Marsh et al., 2022, United States [45]	To improve diabetes management and clinical outcomes among underserved older adults through an integrated model combining community health worker home visits and telemedicine.	Quality improvement study using a pre–post-test design	Community-dwelling older adults with type 1 or 2 diabetes mellitus (*n* = 12) 68.3 ± 3.5	Combined community health worker (CHW) home visits with synchronous telemedicine consultations every two weeks for three months, to deliver patient-centered diabetes education and care. Digital consultations with healthcare providers were embedded within in-home support by CHW.	Rural	Glycosylated hemoglobin (A1C) level ^1^, diabetes self-care behaviors and knowledge, patient satisfaction, healthcare provider satisfaction
Shade et al., 2019, United States [46]	To examine the usability and perceived usefulness of a mobile medication reminder app for supporting medication self-management among rural older adults.	Feasibility study	Community-dwelling older adults taking ≥ 3 prescribed medications (*n* = 15) 69.0 ± 8.4	Mobile medication reminder app to support daily medication self-management over a two-week period, with a six-week follow-up assessment of usability and perceived usefulness. The app provided automated reminders, adherence tracking, and optional self-monitoring features.	Rural	Ease of use of the mobile app, medication adherence, and self-reported confidence, effort, and perceived need for assistance
Gong et al., 2019, China [47]	To develop and contextualize a message-based mHealth intervention to support secondary stroke prevention and self-management in rural, resource-poor settings.	Multi-method intervention	Community-dwelling older adults who have experienced a stroke (*n* = 54) 68.0 ± 9.2	Mobile health intervention delivered structured text and voice messages every two days over three months to support secondary stroke prevention and self-management. Message content and delivery algorithms were integrated into a digital health management system and contextualized to local literacy levels, language, and preferences.	Rural	Feasibility, acceptability, and comprehension of the message-based intervention, and whether participants received and engaged with messages, found them helpful, and understood the content
Nancarrow et al., 2016, Australia [48]	To evaluate the impact of home-based telemonitoring, supported by a telehealth nurse, on self-management, health outcomes, and health service use among older adults with chronic disease in a regional setting.	Mixed-methods longitudinal study	Community-dwelling older adults with at least one chronic disease (*n* = 200) 74.8 ± 8.2	Home-based telemonitoring intervention involves daily monitoring of vital signs via connected devices, with data remotely reviewed by telehealth nurses. Videoconferencing was used to provide tailored clinical advice, reinforce self-management, and respond to abnormal readings.	Regional	Participants’ self-management ^2^ and health outcomes

^1^ A1C reflects average blood glucose over 3 months and was assessed pre- and post-intervention. ^2^ Participants’ understanding of their vital signs, confidence in discussing health with their doctors, engagement in health-promoting behaviors such as improved diet, increased physical activity, and medication adherence, as well as psychological effects like reduced worry and increased reassurance, comfort and confidence using technology, participation in group videoconferences for health literacy, self-management, or social support.

## Data Availability

The original contributions presented in this review are included in the article.

## Supplementary Materials

The following supporting information can be downloaded at https://www.mdpi.com/article/10.3390/ijerph23060713/s1, File S1: Search strategy, File S2: Factors identified as influencing the implementation of technology-enabled care interventions for community-dwelling older adults in RRR settings, mapped to barrier or enabler, CFIR domain and CFIR construct, Table S1: Preferred Reporting Items for Systematic reviews and Meta-Analyses extension for Scoping Reviews (PRISMA-ScR) Checklist.

## Abbreviations

The following abbreviations are used in this manuscript:

RRR	Rural, regional, and remote
CFIR	Consolidated Framework for Implementation Research
