# Supplementary material for: Barriers and Enablers in Implementing Technology-Enabled Care for Older Adults in Rural and Remote Settings: A Scoping Review
Source: Int J Environ Res Public Health. 2026 May 27;23(6):713. doi: 10.3390/ijerph23060713 (PMC13300442; doi:10.3390/ijerph23060713)
Supplement: Supplementary file 1 [file ijerph-23-00713-s001.zip › ijerph-4274131-supplementary.pdf]

Supplementary File S1. Search strategy

Database	Date	Search strategy	Hits
**PUBMED**			
	02-09-2025	((aged[tiab] OR "older people"[tiab] OR "older adult"[tiab]) AND (rehabilitation[tiab] OR "self-management"[tiab] OR enablement[tiab] OR "community-based rehabilitation"[tiab] OR "home-based rehabilitation"[tiab] OR "domiciliary rehabilitation"[tiab])AND ("telehealth"[tiab] OR "tele-health"[tiab] OR "telemedicine"[tiab] OR "tele-medicine"[tiab] OR "digital health"[tiab] OR "remote consultation"[tiab] OR "remote monitoring"[tiab] OR "video consultation*"[tiab] OR "virtual care"[tiab] OR mHealth[tiab] OR eHealth[tiab] OR "mobile health"[tiab]) AND ("community-dwelling"[tiab] OR "living in the community"[tiab] OR "home-based"[tiab] OR "home care"[tiab] OR "domiciliary care"[tiab]))	91
**Scopus**			
	02-09-2025	(TITLE-ABS-KEY(aged OR "older people" OR "older adult") AND TITLE-ABS-KEY(rehabilitation OR "self-management" OR enablement OR "community-based rehabilitation" OR "home-based rehabilitation" OR "domiciliary rehabilitation") AND TITLE-ABS-KEY("telehealth" OR "tele-health" OR "telemedicine" OR "tele-medicine" OR "digital health" OR "remote consultation" OR "remote monitoring" OR "video consultation*" OR "virtual care" OR mHealth OR eHealth OR "mobile health") AND TITLE-ABS-KEY("community-dwelling" OR "living in the community" OR "home-based" OR "home care" OR "domiciliary care") AND LANGUAGE(English)) AND (LIMIT-TO (DOCTYPE,"ar")) AND PUBYEAR > 2014	409
**Medline(Ovid)**			

	02-09-2025	.ti,ab. (aged OR "older people" OR "older adult") AND .ti,ab. (rehabilitation OR "self-management" OR enablement OR "community-based rehabilitation" OR "home-based rehabilitation" OR "domiciliary rehabilitation") AND .ti,ab. ("telehealth" OR "tele-health" OR "telemedicine" OR "tele-medicine" OR "digital health" OR "remote consultation" OR "remote monitoring" OR "video consultation*" OR "virtu al care" OR mHealth OR eHealth OR "mobile health") AND .ti,ab. ("community-dwelling" OR "living in the community" OR "home-based" OR "home care" OR "domiciliary care") Limiters: English, 2014-2025	46
**Web of Science**			
	02-09-2025	TS (aged OR "older people" OR "older adult") AND TS (rehabilitation OR "self-management" OR enablement OR "community-based rehabilitation" OR "home-based rehabilitation" OR "domiciliary rehabilitation") AND TS ("telehealth" OR "tele-health" OR "telemedicine" OR "tele-medicine" OR "digital health" OR "remote consultation" OR "remote monitoring" OR "video consultation*" OR "virtual care" OR mHealth OR eHealth OR "mobile health") AND TS ("community-dwelling" OR "living in the community" OR "home-based" OR "home care" OR "domiciliary care") Limiters: English, 2014-2025	240
**CINHAL**			
	02-09-2025	XB (aged OR "older people" OR "older adult") AND XB (rehabilitation OR "self-management" OR enablement OR "community-based rehabilitation" OR "home-based rehabilitation" OR "domiciliary rehabilitation") AND XB ("telehealth" OR "tele-health" OR "telemedicine" OR "tele-medicine" OR "digital health" OR "remote consultation" OR "remote monitoring" OR "video consultation*" OR "virtual care" OR mHealth OR eHealth OR "mobile health") AND XB ("community-dwelling" OR "living in the community" OR "home-based" OR "home care" OR "domiciliary care")	21

Supplementary File S2. Factors identified as influencing the implementation of technology-enabled care interventions for community-dwelling older adults in RRR settings, mapped to barrier or enabler, CFIR domain and CFIR construct.

**Reference**	**Influencing factor**	**Barrier (B) / Enabler (E)**	**CFIR Domain**	**CFIR Construct**
Marsh et al	Scheduling home visits/telehealth required high flexibility	B	Implementation Process	Doing
Nancarrow et al	Late recruitment shortened participation/incomplete data	B	Implementation Process	Doing
Leverentz et al	Homogeneous sample limited representativeness	B	Implementation Process	Engaging
Shade et al	Guided support (in-person instruction and practice with app)	E	Implementation Process	Engaging
Leverentz et al	Program delivered by developers may limit transferability	B	Implementation Process	Planning
Nancarrow et al	Structured programme and fidelity checklist	E	Implementation Process	Planning
Marsh et al	Short recruitment timeframe & NBN requirement limited recruitment	B	Implementation Process	Planning
Gong et al.	Pilot feedback integration to refine content	E	Implementation Process	Reflecting & Evaluating (Innovation)
Leverentz et al	Participant characteristics (high health literacy)	E	Individuals	Innovation Recipients - Capability
Marsh et al	CHW engagement	E	Individuals	Innovation Recipients - Capability
Gong et al.	Many participants were illiterate or had low reading ability, limiting text effectiveness	B	Individuals	Innovation Recipients - Capability
Gong et al.	Limited technical skills meant some participants could receive calls but could not read texts or navigate phones	B	Individuals	Innovation Recipients - Capability
Gong et al.	Participants had poor memory or cognitive decline, making retention difficult	B	Individuals	Innovation Recipients - Capability

Gong et al.	Multiple pieces of information in a single message were overwhelming	B	Individuals	Innovation Recipients - Capability
Leverentz et al	Limited understanding of conditions/self-management options	B	Individuals	Innovation Recipients - Capability
Leverentz et al	Requirement for tech skills/access may exclude some older/rural	B	Individuals	Innovation Recipients - Capability
Marsh et al	Limited digital literacy requiring CHW support during telemedicine	B	Individuals	Innovation Recipients - Capability
Nancarrow et al	Wide variation in videoconferencing experience required tailored training	B	Individuals	Innovation Recipients - Capability
Shade et al	Limited prior experience with mobile apps could hinder use	B	Individuals	Innovation Recipients - Capability
Shade et al	Participants noted those with memory impairment might struggle	B	Individuals	Innovation Recipients - Capability
Leverentz et al	Motivational approaches (appreciative inquiry, positive psychology)	E	Individuals	Innovation Recipients - Motivation
Leverentz et al	Willingness to engage	E	Individuals	Innovation Recipients - Motivation
Nancarrow et al	Motivated, self-selected participants	E	Individuals	Innovation Recipients - Motivation
Leverentz et al	Time commitment and scheduling conflicts led to declining participation	B	Individuals	Innovation Recipients - Motivation
Marsh et al	Recruitment/retention influenced by willingness/comfort with technology	B	Individuals	Innovation Recipients - Motivation
Nancarrow et al	Telemonitoring may be ineffective for those unwilling/unable to use tech	B	Individuals	Innovation Recipients - Motivation
Nancarrow et al	Personal contact with healthcare providers (videoconferencing)	E	Individuals	Innovation Recipients - Opportunity
Marsh et al	Extensive CHW training & supervision increased workforce demands	B	Inner Setting	Access to Knowledge and Information
Marsh et al	Pilot was labour-intensive and resource-heavy, limiting scalability	B	Inner Setting	Access to Knowledge and Information

Marsh et al	Structured training and supervision	E	Inner Setting	Access to Knowledge and Information
Nancarrow et al	Tailored training and IT support	E	Inner Setting	Access to Knowledge and Information
Leverentz et al	Individualised, person-centred approach	E	Inner Setting	Culture: Recipient-Centeredness
Marsh et al	Strong therapeutic relationship	E	Inner Setting	Relational Connections
Leverentz et al	Coordination with multiple healthcare providers added complexity	B	Inner Setting	Relational Connections
Marsh et al	Interdisciplinary collaboration (CHWs, nurses, physicians, pharmacists)	E	Inner Setting	Relational Connections
Marsh et al	Technology integration (telemedicine platforms, tablets, mobile internet)	E	Inner Setting	Structural Characteristics: Information Technology Infrastructure
Gong et al.	Local dialect and plain language	E	Innovation	Innovation Adaptability
Gong et al.	Repetition and slower playback speed	E	Innovation	Innovation Adaptability
Gong et al.	Optimised timing and frequency aligned with routines	E	Innovation	Innovation Adaptability
Marsh et al	Patient-centred design (home visits + telemedicine)	E	Innovation	Innovation Adaptability
Shade et al	Use of personal mobile devices	E	Innovation	Innovation Adaptability
Gong et al.	Broad/complex message content reduced comprehension	B	Innovation	Innovation Complexity
Gong et al.	Use of professional/non-plain language created confusion	B	Innovation	Innovation Complexity
Leverentz et al	Minor technology delays due to software updates	B	Innovation	Innovation Complexity
Shade et al	Short, flexible programme format	E	Innovation	Innovation Complexity
Shade et al	Accessible and user-friendly technology	E	Innovation	Innovation Complexity
Leverentz et al	Unused optional app features	B	Innovation	Innovation Complexity
Nancarrow et al	Scheduling unusual medication intervals was difficult	B	Innovation	Innovation Complexity

Shade et al	Simplicity of core features (virtual pillbox, reminders, tracking)	E	Innovation	Innovation Complexity
Shade et al	Short set-up and download time	E	Innovation	Innovation Complexity
Shade et al	Premium app version considered expensive	B	Innovation	Innovation Cost
Gong et al.	Some messages not relevant to individual conditions	B	Innovation	Innovation Design
Gong et al.	Messages delivered from multiple numbers led to mistrust	B	Innovation	Innovation Design
Gong et al.	Timing of message delivery inconvenient	B	Innovation	Innovation Design
Gong et al.	Lack of repetition/slow playback limited understanding	B	Innovation	Innovation Design
Leverentz et al	Voice message delivery preferred over text	E	Innovation	Innovation Design
Shade et al	Structured and focused content (one key point per message)	E	Innovation	Innovation Design
Shade et al	Short intervention duration may be insufficient	B	Innovation	Innovation Design
Shade et al	Difficulty navigating app to upload medications	B	Innovation	Innovation Design
Gong et al.	Small font size & unclear interface features reduced accessibility	B	Innovation	Innovation Design
Gong et al.	Some educational features underutilised, possibly due to complexity	B	Innovation	Innovation Design
Gong et al.	Professional verification by doctors/physicians	E	Innovation	Innovation Evidence-Base
Nancarrow et al	Applied a standardised education curriculum	E	Innovation	Innovation Evidence-Base
Nancarrow et al	Daily monitoring with immediate feedback	E	Innovation	Innovation Relative Advantage
Shade et al	Perceived usefulness of the app	E	Innovation	Innovation Relative Advantage
Gong et al.	Trusted sender (consistent phone number)	E	Innovation	Innovation Source
Gong et al.	Participants lived alone or with only spouse, limiting reinforcement	B	Outer Setting	Local Conditions
Gong et al.	Caregivers not always engaged/able to relay messages	B	Outer Setting	Local Conditions

Marsh et al	Lack of stable internet/devices required CHW devices	B	Outer Setting	Local Conditions
Nancarrow et al	Requirement for broadband/NBN restricted eligible participants	B	Outer Setting	Local Conditions
Shade et al	Integration with existing care (telemonitoring data sent to GPs)	E	Outer Setting	Partnerships & Connections

CHW = Community Health Worker; NBN = National Broadband network
